# Midgut malrotation complicated by small bowel obstruction in an 80-year-old woman: A case report

**DOI:** 10.1016/j.ijscr.2019.09.008

**Published:** 2019-09-18

**Authors:** Maha Haqqani, Mani Seetharaman, Richard Teo, Christian Adkisson, Michelle Nessen, Marc Dauer, Peter K. Kim

**Affiliations:** a Albert Einstein College of Medicine, 1300 Morris Park Avenue, Bronx, NY, 10461, United States; b Jacobi Medical Center, Department of Radiology, 1400 Pelham Parkway South, Building 1, 4th Floor, Room 4N69, Bronx, NY, 10461, United States; c Jacobi Medical Center, Department of Surgery, 1400 Pelham Parkway South, Building 1, 5th Floor, Room 510, Bronx, NY, 10461, United States; d Montefiore Medical Center, Department of Surgery, 3400 Bainbridge Avenue, Medical Arts Pavilion, 4th Floor, Bronx, NY, 10467, United States

**Keywords:** Case report, Malrotation, Small bowel obstruction, Ladd procedure

## Abstract

**Introduction:**

Midgut malrotation results from abnormalities in the 270-degree counterclockwise rotation of the midgut around the axis of the superior mesenteric artery during embryological development, and classically presents early in life with symptoms of intestinal obstruction. Nevertheless, adult cases have occasionally been reported.

**Presentation of case:**

An 80-year-old female with no surgical history was brought to our emergency department for acutely altered mental status. On exam, her abdomen was distended and diffusely tender to palpation. Computed tomography (CT) scan of the abdomen and pelvis showed a dilated loop of jejunum with evidence of mesenteric twist concerning for closed-loop small bowel obstruction. The patient was taken for exploratory laparotomy and was found to have Ladd bands and other findings suggestive of intestinal malrotation. A Ladd procedure was performed and the patient remained under observation. She experienced intermittent abdominal distension and bilious nasogastric tube output, but subsequent CT scans revealed no evidence of obstruction. She was discharged following clinical improvement and ability to tolerate a diet.

**Discussion:**

Malrotation of the small bowel exists on a spectrum depending on the embryologic stage during which anomalous rotation occurs. Classic findings on CT imaging (including abnormal mesenteric vasculature, right-sided duodenojejunal junction, whirlpool signs, and left-sided ascending colon) can provide clues to the existence of malrotation.

**Conclusion:**

Although malrotation is rare in adults, clinical and radiologic findings play an important role in the correct diagnosis of adult malrotation for appropriate and timely intervention.

## Highlights

• Intestinal malrotation results from anomalies in embryological midgut rotation.
• Malrotation usually presents early in life, with rare cases reported in adulthood.
• Ladd procedure remains the mainstay of definitive treatment.
• Radiologic findings have a role in early detection and correction of malrotation.

## 1 Introduction

Midgut malrotation is a congenital anomaly resulting from partial or complete failure of the midgut to complete a 270-degree counterclockwise rotation around the axis of the superior mesenteric artery (SMA) during embryological development [1,2]. The incidence is approximately 0.2–0.5% of all live births, with the majority of symptomatic cases presenting in the early weeks of life with obstructive symptoms such as abdominal pain and bilious vomiting [[3], [4], [5]].

Although malrotation is classically a pediatric problem, there have been reported cases of adults presenting with acute intestinal obstruction that demonstrate findings consistent with malrotation. Such cases appear to occur most often in young adults [[1], [2], [3],^6^], and occasionally in the middle-aged [2,4], with very rare instances of elderly patients [5,7]. Here we present the case of an 80-year-old woman who presented with acute-onset small bowel obstruction (SBO) and was found to have features of malrotation both on radiologic imaging and intraoperatively.

This case report was written in line with the Updated Consensus Surgical Case Report (SCARE) guidelines [8].

## 2 Case presentation

An 80-year-old female with no past surgical history was brought into the Emergency Department (ED) of a public teaching hospital with concern for altered mental status. She had a past medical history of atrial fibrillation (for which she was taking apixaban), two prior cerebrovascular accidents, hypertension, insulin-dependent diabetes mellitus, and Stage 3 chronic kidney disease with a baseline serum creatinine of 1.3–1.5 mg/dL.

**Fig. 1:**
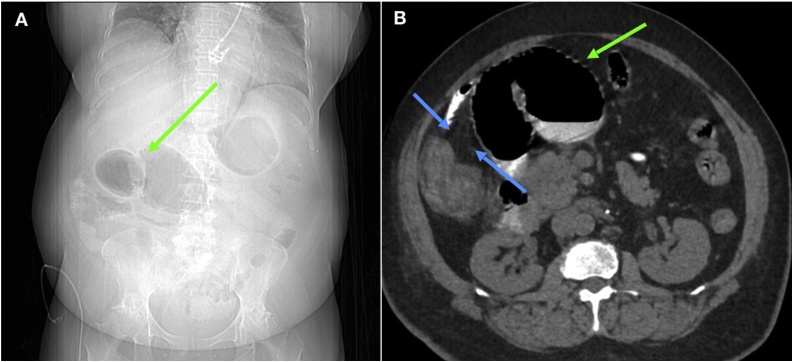
**A.** Scout film of abdomen taken prior to CT imaging upon admission in 2018 showing dilated loops of small bowel (**green arrow**). **B.** CT Abdomen and Pelvis upon admission demonstrating an obstructed and dilated loop of jejunum (**green arrow**). Multiple parallel fine linear densities consistent with Ladd bands (**blue arrows**) are seen extending from ascending colon and cecum.

Upon evaluation, the patient had a Glasgow Coma Score of 8 (E(2)V(2)M(4)) and was unable to provide any history. Initial vitals showed a temperature of 97.8 °F, heart rate of 73 beats/min, blood pressure of 118/65, respiratory rate of 18 breaths/min, and oxygen saturation of 97% on room air. On examination, her abdomen was distended and diffusely tender to palpation. Laboratory results showed an elevated serum creatinine of 5.0 mg/dL consistent with acute kidney injury (AKI). Serum electrolytes, white blood cell count, serum lactate, and coagulation tests were within normal limits. The patient underwent a CT scan of the abdomen and pelvis without contrast, which showed a large dilated loop of proximal jejunum with evidence of mesenteric twist concerning for a closed loop SBO (Fig. 1).

Given the patient’s age and medical comorbidities, her estimated American College of Surgeons National Surgical Quality Improvement Program intraoperative mortality risk was 19%, and the risk of serious complications was 35% [9]. However, due to the acute decline in mental status, AKI, and concerning abdominal exam, emergent surgical intervention was recommended to the next of kin. After obtaining consent, we proceeded with an exploratory laparotomy.

### 2.1 Intraoperative findings

**Fig. 2:**
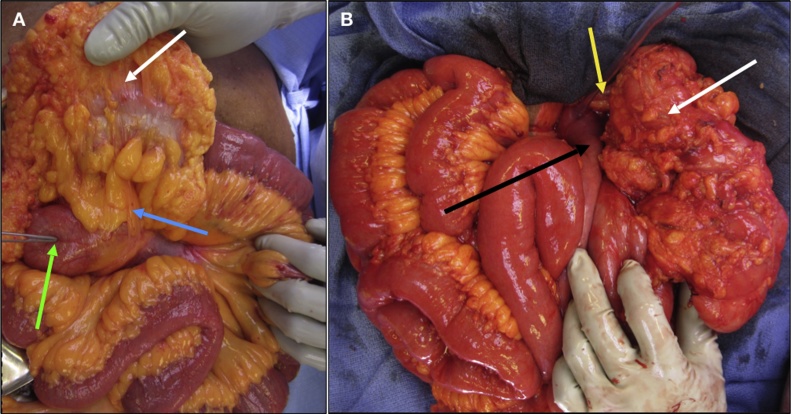
**A.** Intraoperative image taken prior to detorsion and repositioning of abdominal contents, showing dilated loop of jejunum (**green arrow**) seen on preoperative CT imaging. Ladd bands (**blue arrow**) seen extending from cecum (**white arrow**), which is visualized in right *upper* quadrant as opposed to its normal location in the right lower quadrant. **B.** Intraoperative image taken upon repositioning of abdominal contents during Ladd procedure. Duodenum (**black arrow**) now straightened out and located in right hemiabdomen. Cecum (**white arrow**) now visualized in left upper quadrant, along with appendiceal stump secondary to appendectomy (**yellow arrow**).

After intravenous fluid resuscitation and perioperative antibiotics, the patient underwent general anesthesia and intubation. Upon entering the abdomen, we noted several decompressed loops of small bowel, as well as a dilated loop of jejunum densely adherent to the mesentery of the SMA and transverse colon. We also noted twisting of the mesentery and numerous adhesions of the small bowel to the mesentery, retroperitoneum, and surrounding structures. During our lysis of adhesions, we noted a large mesenteric defect through which a loop of jejunum had become trapped, causing the closed loop SBO. Moreover, the cecum was visualized in the right *upper* quadrant instead of its usual anatomical location in the right lower quadrant of the abdomen (Fig. 2A). It became apparent that the adhesions were in fact Ladd bands (Fig. 2A), and that our patient had an intestinal malrotation. After completely lysing the Ladd bands, we then proceeded with a Ladd procedure for malrotation. We first detorsed the mesentery through counterclockwise rotation. The segment of jejunum involved in the internal hernia appeared viable, so no bowel resection was performed. We then mobilized and rotated the cecum and the ascending and transverse colon towards the left, also performing an appendectomy. After straightening out and relocating the duodenum to the right hemiabdomen, the final position of abdominal contents was consistent with that seen after a completed Ladd procedure (Fig. 2B).

### 2.2 Postoperative course

**Fig. 3:**
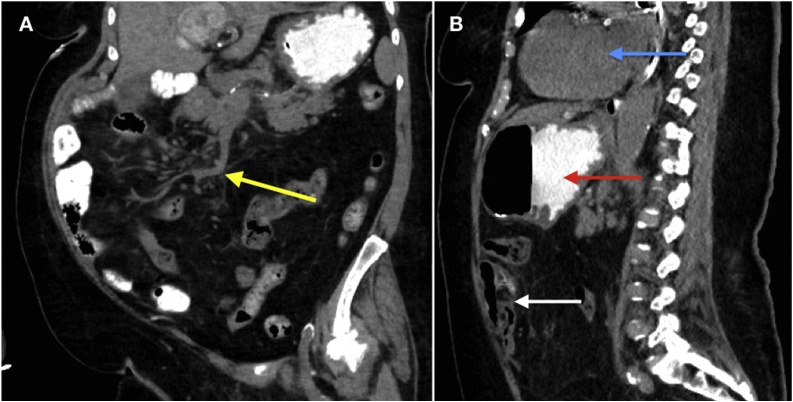
Coronal and sagittal images from non-contrast CT Abdomen and Pelvis obtained during postoperative period. **A.** (Coronal) **Yellow arrow** demonstrating root of mesentery that is no longer twisted. **B.** (Sagittal) New anterior and left hemi-abdomen position of ileocecal junction (**white arrow**). The stomach (**red arrow**) and heart (**blue arrow**) are seen supporting left-sided positioning. Appendix not visualized secondary to appendectomy.

The patient’s postoperative course was accompanied by resolution of AKI but complicated by delirium and prolonged ileus. Initial high-volume bilious output from her nasogastric tube prompted a CT scan that showed no evidence of SBO, and confirmed the new position of the cecum on the left (Fig. 3). The patient was discharged following clinical improvement and ability to tolerate a diet. She was seen in clinic two weeks later and reported feeling well, with no obstructive symptoms.

## 3 Discussion

Midgut malrotation exists on a spectrum with varying presentations depending on the embryologic stage during which anomalous rotation occurs. Normally, midgut lengthening and rotation begins in gestational week 5, with the small intestine starting out as a straight tube and deriving its blood supply primarily from the SMA [10,11]. Between week 5 and weeks 10–11, the gut grows and herniates through the umbilical cord, and the duodenojejunal portion rotates 180° counterclockwise around the axis of the SMA, while the postarterial portion rotates 90° counterclockwise [10,11]. Following this process, the bowel re-enters the abdominal cavity and both segments complete a total 270° counterclockwise turn. The resulting anatomy involves extension of the mesenteric root along the retroperitoneum, from the ligament of Treitz to the cecum [10,11]. Failure of normal embryologic rotation results in a shortened mesenteric root, with the small bowel prone to volvulus around the narrowed SMA axis [11]. Ninety percent of midgut malrotation cases are detected in the first year of life [4,5]. However, our patient presumably remained symptom-free for 80 years, until she finally presented with closed-loop SBO due to acute torsion around the axis of the SMA.

Malrotation has been subclassified into incomplete rotation and non-rotation. During *incomplete* rotation, neither of the two midgut portions rotates more than 180°, and the proximal midgut becomes fixed to the right of the SMA while the cecum is fixed anteriorly. Findings include fibrous peritoneal “Ladd bands” that attach the cecum to the retroperitoneum covering the anterior portion of the duodenum. In *non-rotation*, an under-rotation of both portions leaves the proximal midgut fixed anterior to the right of the SMA and the cecum anterior to the left of the SMA [11].

**Fig. 4:**
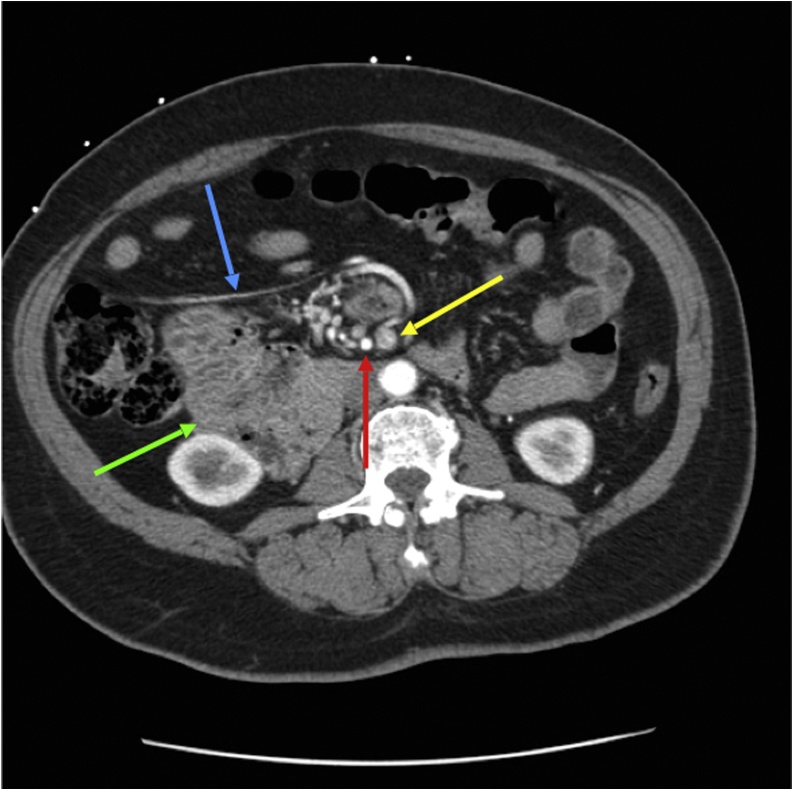
Axial image of prior contrast-enhanced CT Abdomen and Pelvis obtained in 2009, nine years prior to this surgery. Axial image in early arterial phase demonstrating reversal of superior mesenteric artery **(red arrow)** and superior mesenteric vein **(yellow arrow)**. Abnormal linear configuration of possible vessel or Ladd band seen in the right upper quadrant **(blue arrow)**. Third and fourth portions of duodenum (**green arrow)** abnormally seen in the right hemi-abdomen anterior to right kidney and medial to the proximal large bowel, as opposed to their regular configuration in the left hemi-abdomen.

Certain radiologic findings, preferably on CT imaging, can aid in the diagnosis of malrotation. Normally the superior mesenteric vein (SMV) is ventral and to the right of the SMA [7,11]. An abnormal anatomical relationship between the SMA and SMV, and the twisting of small bowel and mesentery around the SMA referred to as the “whirlpool sign” on CT scan [12], should prompt consideration of an underlying malrotation. Other findings include right-sided duodenojejunal junction failing to cross the midline, abnormal cecal position, left-sided colon, and pancreatic uncinate process hypoplasia [11,13]. Upon further review of our patient’s prior CT imaging for another indication in 2009, we observed several findings consistent with malrotation, most notably the abnormal relationship of the SMV to the SMA, and the right-sided duodenum (Fig. 4). If malrotation was recognized by CT scan when asymptomatic, an elective open or laparoscopic Ladd procedure could have been offered to prevent the need for a future emergent operation.

The Ladd procedure for intestinal malrotation, named after the founding American pediatric surgeon William Edwards Ladd (1880–1967), was first described in 1936 [14,15]. The procedure has four components: 1) counterclockwise detorsion of the mesentery along the axis of the SMA, 2) surgical division and dissection of Ladd bands, 3) mobilizing the duodenum to the right and the cecum and colon to the left, and 4) performing a prophylactic appendectomy due to relocation of the cecum [7,10,14]. Both open and laparoscopic approaches may be utilized depending on surgeon skill and experience.

## 4 Conclusion

Intestinal malrotation, while classically a pediatric problem, can present at any age with bowel obstruction or volvulus. Careful review of clinical and radiologic findings could result in early incidental detection and surgical intervention to correct the anatomical defect before it becomes symptomatic and necessitates emergent surgery with increased morbidity and mortality. The Ladd procedure remains the mainstay of definitive treatment for malrotation.

## Funding

There was no source of funding for this case report.

## Ethical approval

This case report was exempt from ethical approval by our institution.

## Consent

Written and signed consent was obtained from the patient at post-hospitalization follow-up visit.

## Author’s contribution

Dr. Haqqani: data collection, chart review, postoperative care of patient, initial draft of manuscript and subsequent revision and editing.

Dr. Seetharaman: Radiology review and analysis, compilation and captioning of CT images.

Dr. Teo: data collection, draft revision and editing.

Dr. Adkisson: data collection, chart review, intraoperative images.

Dr. Nessen: data collection, chart review, postoperative care of patient.

Dr. Dauer: intraoperative decision-making, operative dictation report, draft revision and editing.

Dr. Kim: Initial concept, intraoperative decision-making, draft revision and editing, postoperative care of patient, and approval of final manuscript.

## Registration of research studies

N/A.

## Guarantor

Dr. Peter K. Kim, M.D.

## Provenance and peer review

Not commissioned, externally peer-reviewed.

## Declaration of Competing Interest

The authors have no conflicts of interest to disclose.
